# Case report: Camrelizumab associated with central retinal vein occlusion

**DOI:** 10.3389/fimmu.2022.1025125

**Published:** 2022-11-23

**Authors:** Yixiang Zhan, Weipeng Zhao, Kemin Ni, Zhaoce Liu, Yanjun Su, Xichuan Li, Heng Zhang, Chunze Zhang

**Affiliations:** ^1^ Department of Colorectal Surgery, Tianjin Union Medical Center, Tianjin, China; ^2^ School of Medicine, Nankai University, Tianjin, China; ^3^ Department of Breast Cancer, Key Laboratory of Cancer Prevention and Therapy, Tianjin Medical University Cancer Institute and Hospital, National Clinical Research Center for Cancer, Tianjin, China; ^4^ Department of Lung Cancer, Key Laboratory of Cancer Prevention and Therapy, Tianjin Lung Cancer Center, Tianjin Medical University Cancer Institute and Hospital, National Clinical Research Center for Cancer, Tianjin, China; ^5^ Tianjin Key Laboratory of Animal and Plant Resistance, College of Life Sciences, Tianjin Normal University, Tianjin, China; ^6^ Department of Oncology, Tianjin Union Medical Center, Tianjin, China; ^7^ The Institute of Translational Medicine, Tianjin Union Medical Center of Nankai University, Tianjin, China; ^8^ Tianjin Institute of Coloproctology, Tianjin Union Medical Center, Tianjin, China

**Keywords:** camrelizumab, immunotherapy, case report, vitreous hemorrhage, lung cancer, central retinal vein occlusion

## Abstract

Immunotherapy has revolutionized cancer treatment and become one of the five pillars of cancer therapy. The clinical applications of immunotherapy have been adapted to range from the management of melanoma to most tumor types. As the clinical applications of cancer immunotherapies expand, understanding the treatment-related adverse events of these drugs becomes critical in clinical practice. We report a rare case of ocular immune-related side effects associated with camrelizumab that resulted in vision loss. A 56-year-old male patient was diagnosed with small cell lung cancer. The tumor involved the porta pulmonis and mediastinum upon initial diagnosis; therefore, surgery was not possible. Upon receiving the 10th immunotherapy session with camrelizumab 200 mg, the patient’s visual acuity began to decrease in his right eye and a central retinal vein occlusion. Optical coherence tomography revealed significant cystoid exudation in the macular area and vitreous hemorrhage. The patient underwent vitrectomy, phacoemulsification and intraocular lens implantation after symptom onset. Following surgery, the patient’s vision was limitedly restored. This is the first clinical report in China of central retinal vein occlusion and vitreous hemorrhage associated with anti-PD-1 therapy, ultimately leading to blindness. Although rare, clinical practitioners should be concerned about ocular adverse events associated with anti-PD-1 immunotherapy and develop a high index of suspicion for this possibility since ophthalmic manifestations that are rapidly detected, closely monitored, and appropriately managed are treatable.

## Introduction

Immune checkpoint inhibitors (ICIs), particularly programmed cell death 1 (PD-1) inhibitors, have dramatically shifted the therapeutic armamentarium and outcomes of patients with cancers. The number of reported immune-related adverse events (irAEs) has increased in tandem with the number of cancer patients receiving immunotherapy. These events tend to be mild, treatable, and reversible (1); however, they can be severe in a few cases.

These irAEs can affect any body system at any time, often including the skin, heart, liver, kidneys, etc. However, ophthalmic irAEs are rare, with incidences ranging from 1% to 3% (2, 3). Dry eye and uveitis were the most commonly reported ocular irAEs (4). Mild ophthalmic irAEs have generally been managed successfully using artificial tears and topical steroids without discontinuation of immunotherapy. Some severe rare irAEs, such as Vogt–Koyanagi–Harada (5) syndrome, serous retinal detachment, and retinal vasculitis (6–8), may lead to permanent vision loss if not detected and treated promptly. The retinal vasculitis associated with immune checkpoint inhibitors, in particular, anti-PD-1, has attracted more and more attention recently. Numerous cases of vasculitis following treatment with immunotherapy drugs have been and continue to be reported in published literatures (9–11). This complication can lead to central retinal vein occlusion in both eyes and eventually blindness.

Small cell lung cancer (SCLC) is an aggressive malignancy that is often not diagnosed until the advanced stage (12, 13). Therefore, drugs and radiotherapy are the main treatment options for SCLC. Anti-programmed cell death 1 ligand 1 (PD-L1) inhibitors and platinum-based chemotherapy have been approved as first-line therapies and PD-1 antibodies as third-line therapy setting. With these combinations as the current standard of care, ICIs have helped prolong the overall survival of patients with SCLC (14). The irAEs induced by ICIs treatment in SCLC patients are rarely reported.

In this study, we report a case of camrelizumab-induced central retinal vein occlusion during the treatment of SCLC, a severe ocular irAE associated with PD-1 inhibition. After long-term immunotherapy, this patient presents with diminished vision in his right eye. The optical coherence tomography (OCT) revealed cystoid macular edema in the right eye. He underwent vitrectomy, phacoemulsification, and intraocular lens implantation until B-scan ultrasonography demonstrated vitreous opacities caused by massive vitreous hemorrhage. However, his visual recovery in the right eye was poor, with only weak light perception at 1 year after the operation. This case demonstrates the importance of increased awareness of severe ophthalmic irAEs and limiting potentially dangerous adverse events.

## Case description

A 56-year-old male patient initially received the treatment of PD-1 inhibitor camrelizumab after he was diagnosed with small cell lung cancer (SCLC) for a year and a half and revealed distant lymph node metastasis for 1 year. This patient was a social drinker and a 70-pack-year smoker until 2015. His family history was not notable for SCLC. Anticoagulants and thrombolytics were not administered during cancer treatment. This patient denied eye diseases and reported no long-standing history of hypertension, diabetes, and coronary artery disease when diagnosed with lung cancer.

The tumor involved the porta pulmonis and mediastinum at initial diagnosis; therefore, surgery was not possible. The patient received five chemotherapy treatments with IP (irinotecan, cis-platinum) and prophylactic cranial irradiation between April 2018 to May 2019. When positron emission tomography–computed tomography showed metastasis in the left iliac region, the patient was switched to EN (irinotecan and nedaplatin) chemotherapy and received radiation therapy in the left iliac region. After five cycles of the EN regimen, the patient developed the side effect of severe myelopoiesis inhibition and showed a new space-occupying lesion in the upper lobe of the right lung.

**Figure 1:**
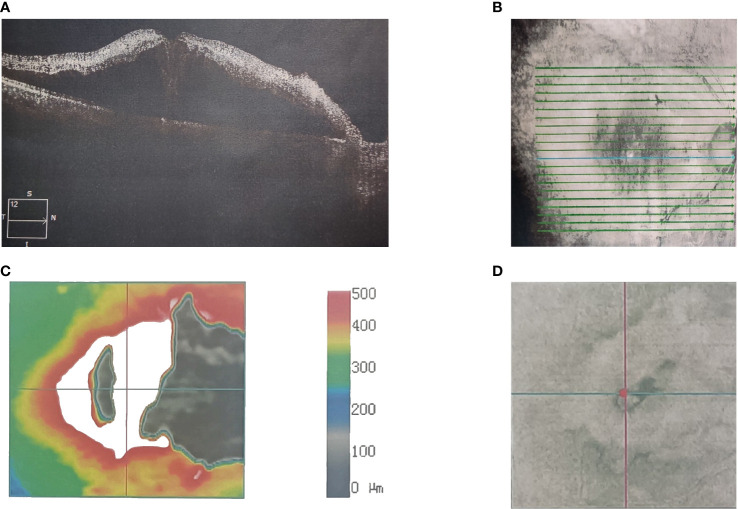
A 56-year-old Asian male with lung cancer and visual acuities (right 20/40). Optical coherence tomography scanning shows the **(A)** microstructure of the macular region of the right eye. **(B)** Scan range of the macular area of the right eye. **(C)** Topographic map of the macular region of the right eye. **(D)** Scan coordinates for the macular region of the right eye.

**Figure 2:**
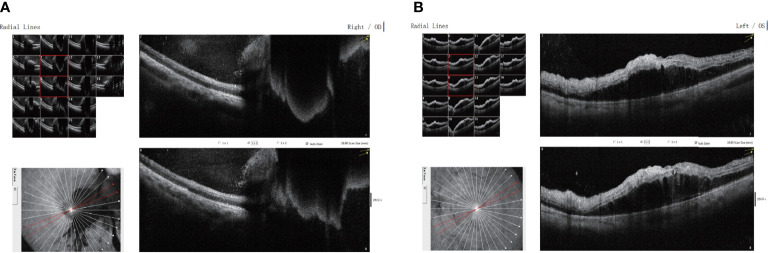
Binocular macular optical coherence tomography scanning through the **(A)** right and **(B)** left eyes showing disordered, uneven signals in the right macular region, with significant fluid accumulation below the right eye and a small amount of fluid bulge below the fovea of the left eye.

Treatment with the PD-1 inhibitor camrelizumab (200 mg, every 3 weeks) was then initiated for the patient. After 10 rounds of immunotherapy, the chest CT showed a significant reduction in the size of the space-occupying chest lesions and number of enlarged mediastinal lymph nodes. The patient noticed diminution of vision in the right eye at 6 days after receiving the 10th camrelizumab immunotherapy with a visual acuity of 20/40, without any vision loss abnormalities in the left eye. Cranial CT did not reveal any intracranial metastasis. He did not immediately notify the oncologist about this symptom. The fundus examination of the right eye revealed scattered patch-shaped intraretinal hemorrhages in all quadrants and cotton-wool exudates, indicative of central retinal vein occlusion. The OCT revealed cystoid macular edema in the right eye (**Figure 1**). The patient received oral tanakan (ginkgo biloba extract) and was subcutaneously injected with the compound anisodine and additional camrelizumab immunotherapy, but the eye visual acuity worsened to 20/200. A second OCT scan showed a fluid bulge below the fovea in the left eye and a disordered, uneven signal in the right macular region with significant fluid accumulation underneath (**Figure 2**).

**Figure 3:**
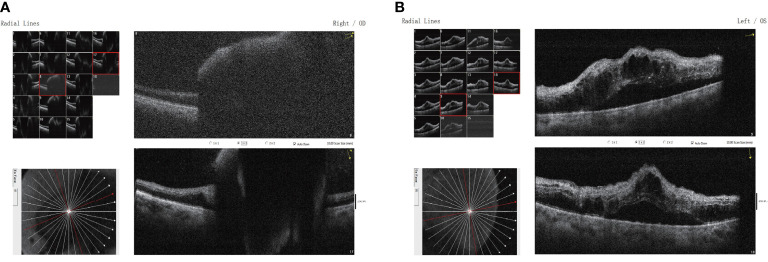
Binocular macular optical coherence tomography scanning through the **(A)** right and **(B)** left eyes showing a subretinal lesion of the right eye with elevated pigment epithelium and neuroepithelium in the macular region and mild cystic macular edema in the left eye.

**Figure 4:**
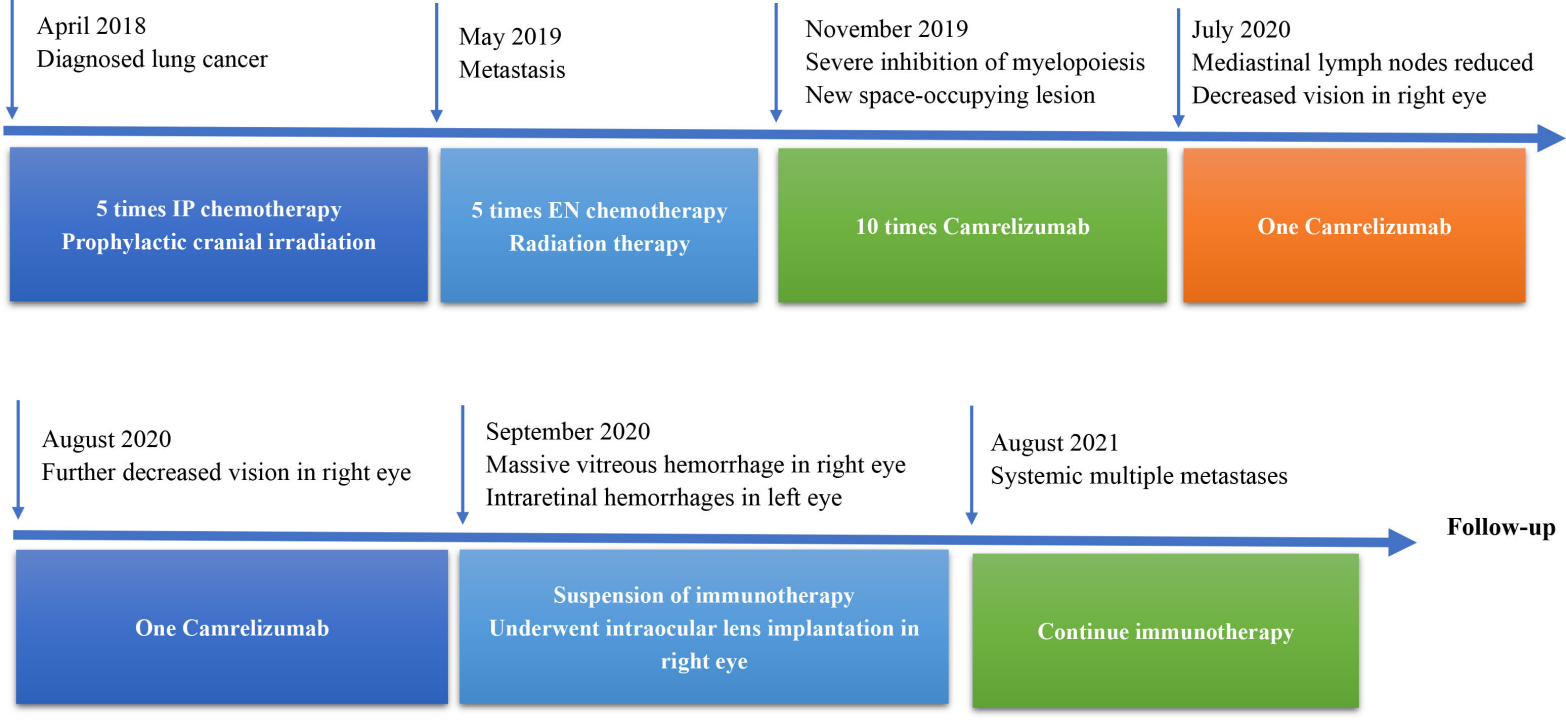
Timeline therapy administration from the episode of care. IP, irinotecan cis-platinum; EN, irinotecan, nedaplatin.

The patient developed severe vision loss in the right eye and could hardly see anything at 2 days after receiving camrelizumab immunotherapy once again. The retina and the macula of the right eye were not visible on fundus examination due to a massive vitreous hemorrhage. Simultaneously, cystoid macular edema and scattered patch-shaped intraretinal hemorrhages were detected in the left eye (**Figure 3**). The B-scan ultrasonography revealed vitreous opacities and some organic compounds in the right eye. Hence, the patient underwent vitrectomy, phacoemulsification, and intraocular lens implantation in his right eye. The immunotherapy was stopped, and the patient’s symptoms in both eyes improved. The patient was closely followed-up, and visual recovery in the right eye was poor. His visual acuity was 20/200 in the right eye at 1 year after the operation. The patient provided written informed consent for the publication of this case, and the timeline of therapy administration from the episode of care is shown in **Figure 4**.

## Discussion

The large-scale application of ICIs has greatly improved the prognosis of patients with terminal cancer, which has been an exciting development in recent years (15–17). Despite the fact that SCLC is usually sensitive to chemotherapy and radiotherapy, a large proportion of patients relapse with metastases at other sites after receiving initial treatment (18). Unfortunately, very few drugs have been approved as effective second-line treatments for SCLC. The introduction of PD-1/PD-L1 inhibitors has changed the treatment patterns and improved the survival of patients with SCLC.

Immunotherapy-related cutaneous toxicities are the most common irAEs and are extremely common in cancer patients treated with PD-1/PD-L1 inhibitors (19). Meanwhile, the incidence of ophthalmic irAEs, commonly no more than 1%, is considered low (20). However, according to a decade-long study from the Mayo Clinic, although ophthalmic irAEs are rare, such as dry eye, uveitis, singular cases of ptosis, and binocular diplopia, they could be more common than previously reported (3) and typically occur in conjunction with systemic irAEs (21).

Most irAEs are triggered by cytotoxic CD4+/CD8+ T cell activation (22). Blockage of the PD-1 pathway causes the immune cells to switch to a proinflammatory Th1/Th17 state (23). Furthermore, the direct binding of antibody against immune checkpoint inhibitors can activate the immune complement system (24). Accordingly, this proinflammatory state will cause central retinal vein occlusion, resulting in retinal hemorrhage. This drug-induced abnormal state leads to a systemic inflammatory response that is more likely to result in bilateral rather than unilateral fundus lesions.

Vasculitis and central retinal vein occlusion can occur as a paraneoplastic phenomenon with malignancy, and these phenomena can alleviate with surgical resection or drug therapy. In our report, after receiving immunotherapy treatment twice again, the vision of the right eye was further reduced. The patient’s ocular symptoms appeared while receiving immunotherapy treatment, and the symptoms in the left eye alleviated after stopping immunotherapy. Therefore, we consider that the symptoms of the patient that we reported were secondary to camrelizumab-induced vasculitis and not caused by other factors such as hypertension, hyperglycemia, hyperlipidemia, obesity, carotid artery obstructive disease, hemorheology abnormalities, thrombosis, blood viscosity, etc. (25).

Ophthalmic irAEs typically occur weeks to months after ICI treatment and occasionally even after cessation of treatment. In general, these irAEs are mild and can be cured by topical and periocular treatments. In our case, the patient was not actively receiving initial treatment when he noticed the impaired vision, and the ophthalmologists did not associate the diminution of vision with immunotherapy. Therefore, after the patient received two additional cycles of immunotherapy, the retinal ischemia was further aggravated. This indicates that clinicians should be aware of the overall presentation of ophthalmic irAEs so that, when serious ophthalmic complications occur, immunotherapy is immediately stopped in a multidisciplinary consultation with oncologists.

There are numerous uncertainties regarding the correct management of potential PD-1 inhibitor toxicities, which must be closely monitored. We report a case of camrelizumab-induced central retinal vein occlusion during SCLC treatment, which eventually resulted in blindness in the right eye due to retinal hemorrhage. Regretfully, due to picture quality and quantity limitations, we did not show the results of the B-scan ultrasonography for this patient’s right eye. Considering the severity of this event, this rare complication must be seriously considered. To the best of our knowledge, there have been no previous reports of retinal venous occlusion caused by camrelizumab. In conclusion, further research is warranted to standardize the management of ophthalmic irAEs and determine the toxicity pathogenesis to maximize the benefits of treatment.

## Data availability statement

The original contributions presented in the study are included in the article. Further inquiries can be directed to the corresponding authors.

## Ethics statement

Written informed consent was obtained from the individual(s) for the publication of any potentially identifiable images or data included in this article.

## Author contributions

CZ and HZ provided study concept, design, and project execution. YZ drafted the manuscript and included the intellectual content and the revisions from KN and ZL. KN processed the images. HZ, WZ, and YS participated in the whole process of patient diagnosis and treatment. XL and ZL contributed to writing of the manuscript. All authors contributed to the article and approved the submitted version.

## Funding

This work was supported by the Natural Science Foundation of Tianjin (no. 21JCYBJC00180), Key R&D Projects in the Tianjin Science and Technology Pillar Program (no. 19YFZCSY00420 and no. 18ZXDBSY00040), Tianjin Key Medical Discipline (Specialty) Construction Project (no. TJYXZDXK-044A), the National Natural Science Foundation of China (no. 81872236 and no. 81472183), and Scientific Research Project of Tianjin Educational Committee (no. 2017ZD11).

## Acknowledgments

We thank the patient’s relatives for providing permission to share the patient’s information. We thank doctor Ziyi Dong for his guidance in ophthalmology during the revision.

## Conflict of interest

The authors declare that the research was conducted in the absence of any commercial or financial relationships that could be construed as a potential conflict of interest.

## Publisher’s note

All claims expressed in this article are solely those of the authors and do not necessarily represent those of their affiliated organizations, or those of the publisher, the editors and the reviewers. Any product that may be evaluated in this article, or claim that may be made by its manufacturer, is not guaranteed or endorsed by the publisher.
